# Angiopoietin-like protein 3: a novel potential biomarker for nephrotic syndrome in children

**DOI:** 10.3389/fped.2023.1113484

**Published:** 2023-05-17

**Authors:** Fujie Wen, Junchao Liu, Rufeng Dai, Sha Hong, Baowei Ji, Jiaojiao Liu, Jun Zhang, Xinli Han, Qianying Lv, Jialu Liu, Qian Shen, Hong Xu

**Affiliations:** ^1^ Department of Nephrology, Children's Hospital of Fudan University, Shanghai, China; ^2^ Department of Traditional Chinese Medicine, Children's Hospital of Fudan University, Shanghai, China; ^3^ Department of Rheumatology, Children's Hospital of Fudan University, Shanghai, China

**Keywords:** angiopoietin-like protein 3, nephrotic syndrome, biomarker, proteinuria, children

## Abstract

**Background:**

Angiopoietin-like 3 (ANGPTL3) is a secretory glycoprotein. It has been demonstrated that ANGPTL3 level was upregulated in minimal change nephrotic syndrome (MCNS) kidney tissues. Subsequently, our group found that ANGPTL3 level was closely correlated with nephropathy *in vivo and in vitro*. Hence, whether ANGPTL3 level could be correlated with the proteinuria level, and assessment of disease severity of nephrotic syndrome (NS) remained to be investigated. This study aimed to analyzed the correlation between the levels of ANGPTL3 in serum and urine of patients with nephrotic syndrome and proteinuria, and assessed the severity of the patients' disease. In future clinical translation, the level of ANGPTL3 in serum, urine will be used as a biomarker to better predict the development of nephrotic syndrome.

**Methods:**

A total of 200 NS patients and 80 healthy controls (age, 1–18 years) were admitted to our institution between 2021 and 2022. The etiology of NS included primary nephrotic syndrome (PNS, *n* = 144) and NS with other causes (*n* = 56). A total of 280 serum samples and 244 urinary samples were collected to determine ANGPTL3 level using enzyme-linked immunosorbent assay (ELISA).

**Results:**

Serum ANGPTL3 and urinary ANGPTL3/Cre were remarkably elevated in NS patients compared with those in healthy controls. Furthermore, serum ANGPTL3 and urinary ANGPTL3/Cre were significantly correlated with proteinuria level. Additionally, multivariate linear regression analysis demonstrated that serum ALB was independently correlated with serum ANGPTL3 and PRO/CR was independently correlated with urinary ANGPTL3/Cre in NS patients.

**Conclusion:**

Serum ANGPTL3 and urinary ANGPTL3/Cre showed a promising performance in the diagnosis of NS, and served as novel potential noninvasive biomarkers to assess disease severity of NS. Further exploration of the role of ANGPTL3 level may shed a new light on the treatment of NS.

## Introduction

Nephrotic syndrome (NS) is defined by the presence of nephrotic range proteinuria, edema, hyperlipidemia, and hypoalbuminemia (1). The glomerulus is a highly specialized structure in the kidney that ensures selective ultrafiltration of plasma, so that essential proteins are retained in the blood (2). The significance of glomerulus is underscored by a series of kidney diseases, such diabetes mellitus, systemic lupus erythematosus, and familial focal segmental glomerulosclerosis. Their common denominator is glomerular dysfunction, involving a massive loss of protein in the urine (2). However, the proteinuria was the core clinical presentation and closely associated with the podocyte damage and the progression of kidney dysfunction (3). Glomerular podocyte injury may induce glomerular filtration membrane injury and proteinuria. In addition, some patients are insensitive to glucocorticoids and immunosuppressants, and their proteinuria cannot be relieved until the kidney function quickly progresses to kidney failure. Therefore, it is particularly crucial to find a new biomarker to better monitor disease severity of NS and future use of this molecule as a target to better treat patients with renal podocyte-damaging proteinuria to minimize its deleterious consequences.

Angiopoietin-like 3 (ANGPTL3) is a secretory glycoprotein with a molecular weight of 70 kDa, and belongs to the angiopoietin-like (ANGPTL) family members. In 1999, the ANGPTL3 was discovered (4). Thereafter, several studies have reported the role of ANGPTL3 level in tumor, coronary heart disease, metabolic disorders, dyslipidemia, etc. (5–14). However, few studies have concentrated on the role of ANGPTL3 in NS, and we first discovery that ANGPTL3 was upregulated in minimal change nephrotic syndrome (MCNS) kidney tissues, which clarified the importance of ANGPTL3 in the pathogenesis of podocyte injury and nephropathy proteinuria (15). Moreover, in order to use ANGPTL3 as a better target molecule in clinical patients with renal podocyte-injurious proteinuria, we prepared antibodies against different structural domains of ANGPTL3 and validated its effects on podocytes and proteinuria in animal and in cellular assays. As Han XL et al. (16) prepared an antibody against ANGPTL3 coil-coiled domain (ANGPTL3-CCD) and investigated the protective effect of anti-ANGPTL3-CCD antibody on the Adriamycin (ADR)-induced nephropathy in rats. Another study prepared an antibody against ANGPTL3 fibrinogen-like domain (ANGPTL3-FLD) in a mouse model of ADR-induced nephropathy (17). ANGPTL3 was closely correlated with nephropathy *in vivo and in vitro*, knockdown of ANGPTL3 or anti-ANGPTL3 monoclonal antibodies showed significant protective effects on rats with ADR-induced nephropathy (16–20). These studies suggest that ANGPTL3 is a promising and important target molecule for the treatment of patients with proteinuria in nephropathy. Previous studies have shown that ANGPTL3 is closely associated with podocyte injury (20, 21), the occurrence of proteinuria *in vitro* and *in vivo*, and a significant increase in ANGPTL3 secretion from the damaged podocytes may enter the blood and urine. Therefore, we hypothesized that elevated levels of ANGPTL3 in the blood and urine of patients with nephrotic syndrome may represent the severity of podocyte injury, as well as proteinuria. In addition, we analyzed 11 patients with nephrotic syndrome who had both serum, urine, and renal tissues, and our analysis revealed that the expression levels of ANGPTL3 in serum, urine, and glomeruli were strongly correlated with the severity of podocyte injury (to be published). Therefore, we analyzed the correlation between the levels of ANGPTL3 in serum and urine of patients with nephrotic syndrome and proteinuria, and assessed the severity of the patients' disease. In future clinical translation, the level of ANGPTL3 in serum, urine will be used as a biomarker to better predict the development of nephrotic syndrome.

## Patients and methods

### Study participants

This study involved 200 NS patients and 80 healthy children as normal control group (age, 1–18 years) who were admitted to the Children's Hospital of Fudan University (Shanghai, China) between 2021 and 2022. Patients had primary NS (PNS, *n* = 144) and NS with other causes (*n* = 56), such as IgA nephropathy (IgAN), Henoch–Schonlein purpura (HSP), and lupus nephritis (LN), the number ratio of males/females = 134/66, age in months: 90(50.25, 141.75). Healthy control group of 80 cases, number ratio of male/female = 51/29, age in months: 81.29 ± 35.64. A total of 280 serum samples and 244 urinary samples were eventually collected after applying the inclusion and exclusion criteria.

The inclusion criteria were as follows: (1) the glomerular diseases included PNS and NS with other causes, such as IgAN, HSPGN, and LN. The glomerular diseases were defined based on criteria presented by the International Study for Kidney Diseases in Children. (2) Glomerular filtration rate (GFR) was estimated at the time of enrollment in patients with glomerular diseases who received corticosteroids and rituximab (eGFR ≥ 90 ml/min/1.73 m^2^). (3) Subjects in the control group were healthy children (no kidney injury/proteinuria, etc.). (4) Individuals who aged 1–18 years and signed the informed consent form by themselves or their legal representatives.

The exclusion criteria were as follows: (1) patients with severe respiratory or gastrointestinal infection, severe hepatic dysfunction, coronary heart disease, or kidney dysfunction. (2) Patients with a history of chronic kidney disease, including chronic dialysis or kidney transplantation. (3) Patients with metabolic syndrome, neoplasia, primary hypothyroidism, homozygous familial hypercholesterolemia (FH), obesity, or congenital leptin deficiency. (4) Patients who suffered from severe congenital malformation or serious functional dysfunction. (5) No sign of informed consent form.

## Sample collection

### Serum

All study subjects underwent blood test in the morning after an overnight fasting. Blood was sampled from the arm vein, collected by yellow-capped serum tubes, stored for 30 min at room temperature, and centrifuged for 10 min at 2,000×*g* (4°C) to obtain the serum. The serum samples were stored at −80°C until further analysis, and multiple thaw/freeze cycles were avoided.

### Urine

Mid-stream urine samples were collected in the morning using sterile containers, and centrifuged at 2,000  ×  g for 10 min at 4 °C. The supernatant was sub-packaged into 1.5 ml EP tubes, and was then concentrated in a vacuum concentrator (Savant SpeedVac DNA130) to a final volume of 0.5 ml. All samples were kept frozen at −80°C for experiments, and multiple thaw/freeze cycles were avoided.

### Measurement of serum, urinary ANGPTL3, and urinary creatinine

All indicators were measured in the same serum and urine sample, and the indicators included serum ANGPTL3, urinary ANGPTL3, urinary creatinine (Cre), urinary protein (PRO), urinary protein/creatinine (PRO/Cre), 24 h quantitative urinary protein (24 h UPRO), serum ALB, total cholesterol (TC), and triglyceride (TG). Serum ANGPTL3 level was measured using a human ANGPTL3 simple step ELISA kit (Cat. No. ab254510; Abcam, Cambridge, UK). Serum samples at an optimal dilution were added to a microplate with the mixture of capture antibody and detection antibody (cocktail) for incubation, followed by addition of substrate for color development, and optical density (OD) was measured at 450 nm. Urinary ANGPTL3 level was measured using a Quantikine ELISA Human Angiopoietin-like 3 Immunoassay kit (R&D DANL 30; R&D Systems, Inc., Minneapolis, MN, USA). The concentrated urine using a vacuum concentrator was applied for detection of urinary ANGPTL3 level. Serum and urinary samples were measured in duplicate. To correct the influence of concentrated urine on ANGTPL3 level, the urinary Cre level was measured using a creatinine assay kit (C011-2-1; Nanjing Jiancheng Co., Ltd., Nanjing, China). Urinary ANGPTL3 level was adjusted by urinary Cre excretion. The other indicators, including 24 h UPRO, PRO, PRO/Cre, serum ALB, TC and TG were measured by an autoanalyzer in the Department of Clinical Laboratory, Children's Hospital of Fudan University.

### Clinical data collection

Clinical data, such as sex, age, body mass index (BMI), clinical symptoms, and urinary and blood biological parameters were collected in the first or second day of hospitalization.

### Statistical analysis

Statistical analysis was performed using GraphPad Prism 9.0 (GraphPad Software Inc., La Jolla, CA, USA), SPSS 26.0 (IBM, Armonk, NY, USA) software. Shapiro-Wilk test was used to evaluate the normality of data. When describing, quantitative data are described by mean ± standard deviation if they conform to normal or approximately normal distribution; quantitative data with non-normal distribution are described by median (interquartile range) (IQR). Student's *t*-test was utilized to analyze the differential expression between two groups with normally distributed data, while Mann–Whitney *U*-test was adopted for comparing abnormally distributed data between two groups. In this study, the Mann-Whitney U test was used to compare the indicator of conformity to non-normal distribution between NS and Control 2 groups. For comparison of three or more groups, analysis of variance (ANOVA) was used for normally distributed data, and the Kruskal–Wallis test was applied for comparing abnormally distributed quantitative data. In this study, comparing serum ANGPTL3 and urinary ANGPTL3/Cre levels between 3 groups with different degrees of proteinuria with Kruskal–Wallis tests. Differences in sex ratio were tested by the Chi-square test. Correlation analysis was performed to assess the correlation between ANGPTL3 and each variable (Age, BMI, 24 h UPRO, PRO/CR, serum albumin, TC, and TG), spearman correlation analysis was used to analyze the correlation, the spearman correlation coefficient *r* was used to show the correlation results. The multivariate linear regression analysis was employed to identify variables that were independently associated with ANGPTL3. Area under the curve (AUC) and *P*-value were calculated to determine the role of ANGPTL3 level in the diagnosis of NS. All data were presented as mean ± standard error. *P* < 0.05 was considered statistically significant.

## Results

### Subjects’ characteristics

Subjects' baseline characteristics are presented in Table 1. As expected, patients with NS had higher BMI and serum TC & TG levels, while lower serum ALB levels than controls. However, sex and age were comparable between NS and control groups.

**Table 1:** The baseline characteristics and laboratory data of all patients.

Variables	NS (*n* = 200)	Control (*n* = 80)	*P*
	PNS (*n* = 144), others (IgAN, HSPGN, LN, *n* = 56)		
Sex (male/female, *n*)	134/66	51/29	0.604
Ages (month)	90.00 (50.25, 141.75)	81.29 ± 35.64	0.0687
BMI (kg/m²)	18.00 (16.00, 21.00)	15.80 (14.90, 16.64)	<0.0001
PRO (−) (serum/urinary, n)	50/38	80/80	–
PRO (+) (serum/urinary, n)	150/126	–	–
PRO/CR (mg/mg)	2.29 (0.24, 9.48)	–	–
NA (serum/urinary, n)	16/15	–	–
(0–<0.2) (serum/urinary, n)	40/31	–	–
(0.2–<2) (serum/urinary, n)	49/44	–	–
(≥ 2) (serum/urinary, n)	95/74	–	–
24 h UPRO (g/L)	1.35 (0.34, 4.73)	–	–
Serum ALB (g/L)	31.00 (19.01, 39.40)	43.83 (42.10, 45.16)	<0.0001
TC (mmol/L)	6.66 (4.60, 11.00)	4.21 (3.38, 4.92)	<0.0001
TG (mmol/L)	1.97 (1.21, 3.34)	0.14 (0.07, 0.76)	<0.0001

The values were indicated as the mean ± standard deviation or median (interquartile). NS vs. control were tested by Mann–Whitney *U*-test. Sex ratios were tested by the *χ*^2^ test. *n*, number; NA, not available. *P*: NS vs. control.

### ANGPTL3 level was elevated in the serum and urine samples of NS patients

**Figure 1:**
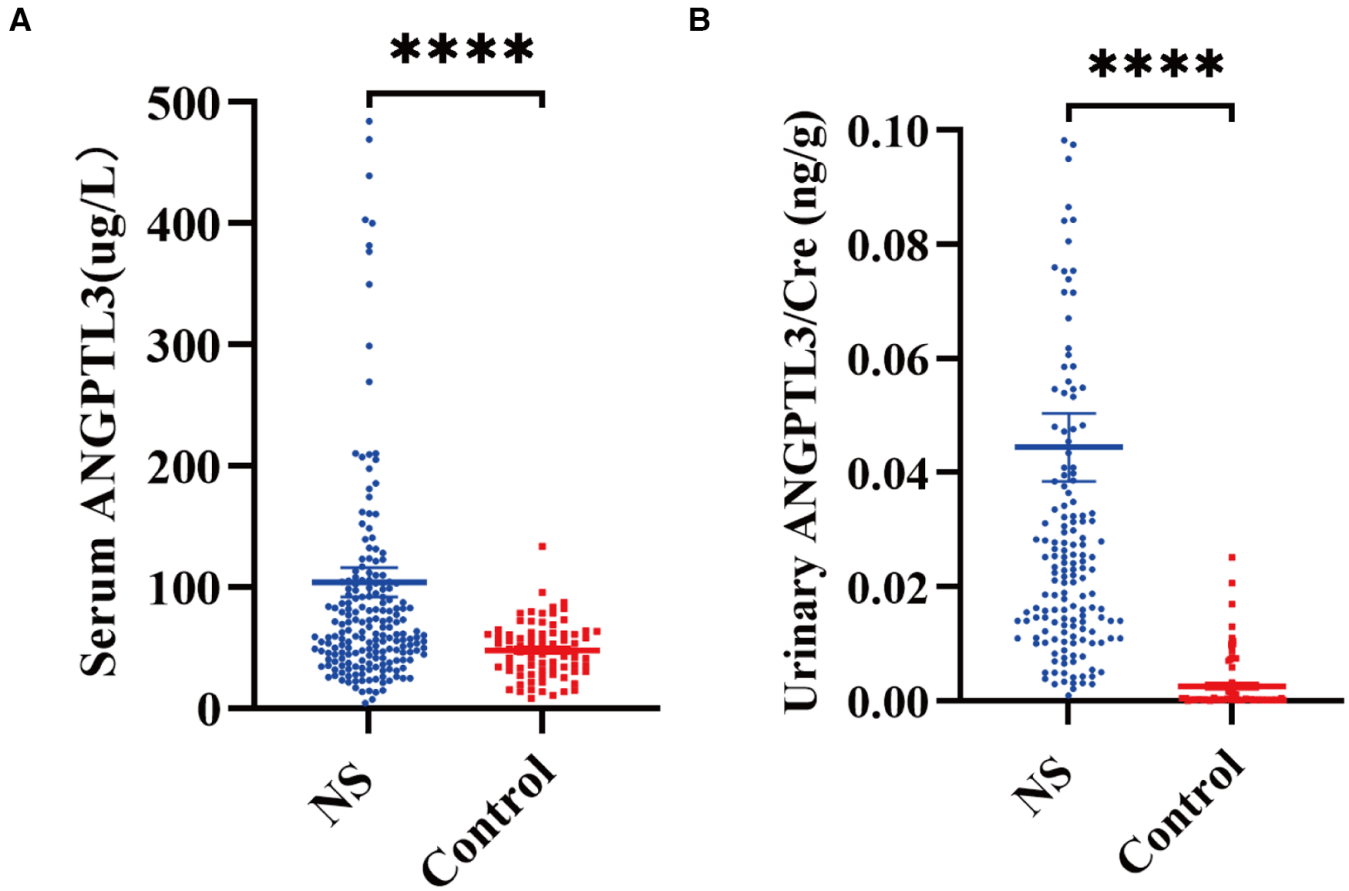
Comparison of ANGPTL3 level in the serum and urine of NS patients and controls. (**A**) Serum ANGPTL3 level in NS and controls. NS, *n* = 200; Control, *n* = 80. (**B**) Urinary ANGPTL3/Cre level in NS and controls. NS, *n* = 164; Control, *n* = 80. Using Mann–Whitney *U*-test, ****P < 0.0001.

It was attempted to determine ANGPTL3 level in the serum and urine samples of NS patients and aged-matched controls by ELISA. By using the Mann–Whitney *U*-test, the results demonstrated that NS patients had increased ANGPTL3 level in both serum and urine samples. Serum ANGPTL3: 62.113 (41.529, 102.761) µg/L vs. 47.466 (31.133, 61.650) µg/L, *P* < 0.0001; urinary ANGPTL3/Cre: 0.0238 (0.0127, 0.0428) ng/g vs. 0.0003 (0.0002, 0.0016) ng/g, *P* < 0.0001] (Figure 1).

### The diagnostic value of serum ANGPTL3 and urinary ANGPTL3/Cre for NS

**Figure 2:**
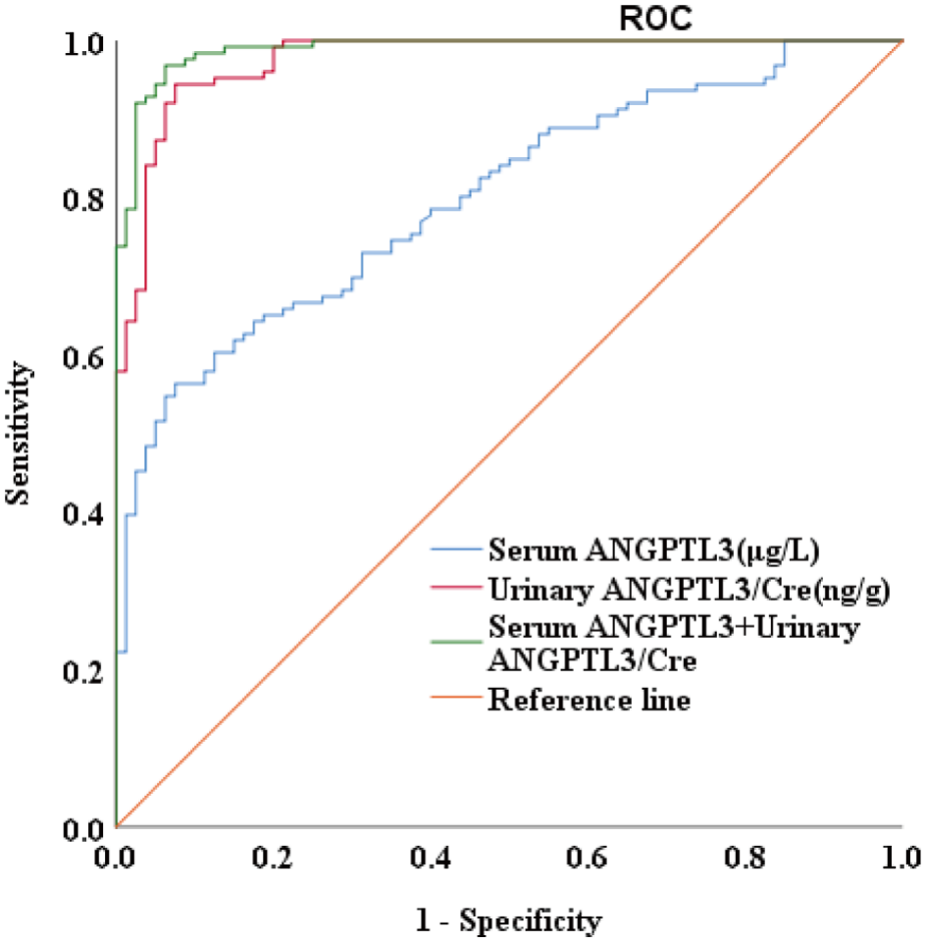
The diagnostic value of serum ANGPTL3 and urinary ANGPTL3/Cre for NS. The AUC was 0.800 for serum ANGPTL3, 0.975 for urinary ANGPTL3/Cre, and 0.989 for combined serum ANGPTL3 and urinary ANGPTL3/Cre, *P *= 0.000.

To evaluate the diagnostic value of ANGPTL3 for NS, the receiver operating characteristic (ROC) curve analysis was performed on 126 PRO-positive NS patients via measurement of both serum and urinary ANGPTL3 levels. Serum ANGPTL3 level had area under the curve (AUC) value of 0.800, *P*-value of 0.000, and cutoff value of 78.940 µg/L, sensitivity was 56.3%, specificity was 92.5%. The AUC of urinary ANGPTL3/Cre was 0.975, with *P*-value of 0.000 and cutoff value of 0.0101 ng/g, sensitivity was 94.4%, specificity was 92.5%. The combination of serum ANGPTL3 and urinary ANGPTL3/Cre improved diagnostic value for NS with AUC of 0.989 and *P*-value of 0.000 (Figure 2). The above-mentioned results indicated that serum and urinary ANGPTL3 may be a helpful biomarker that can distinguish NS patients with urine protein from controls.

### Serum ANGPTL3 and urinary ANGPTL3/Cre were correlated with the degree of proteinuria, and reflected the disease severity of NS

**Figure 3:**
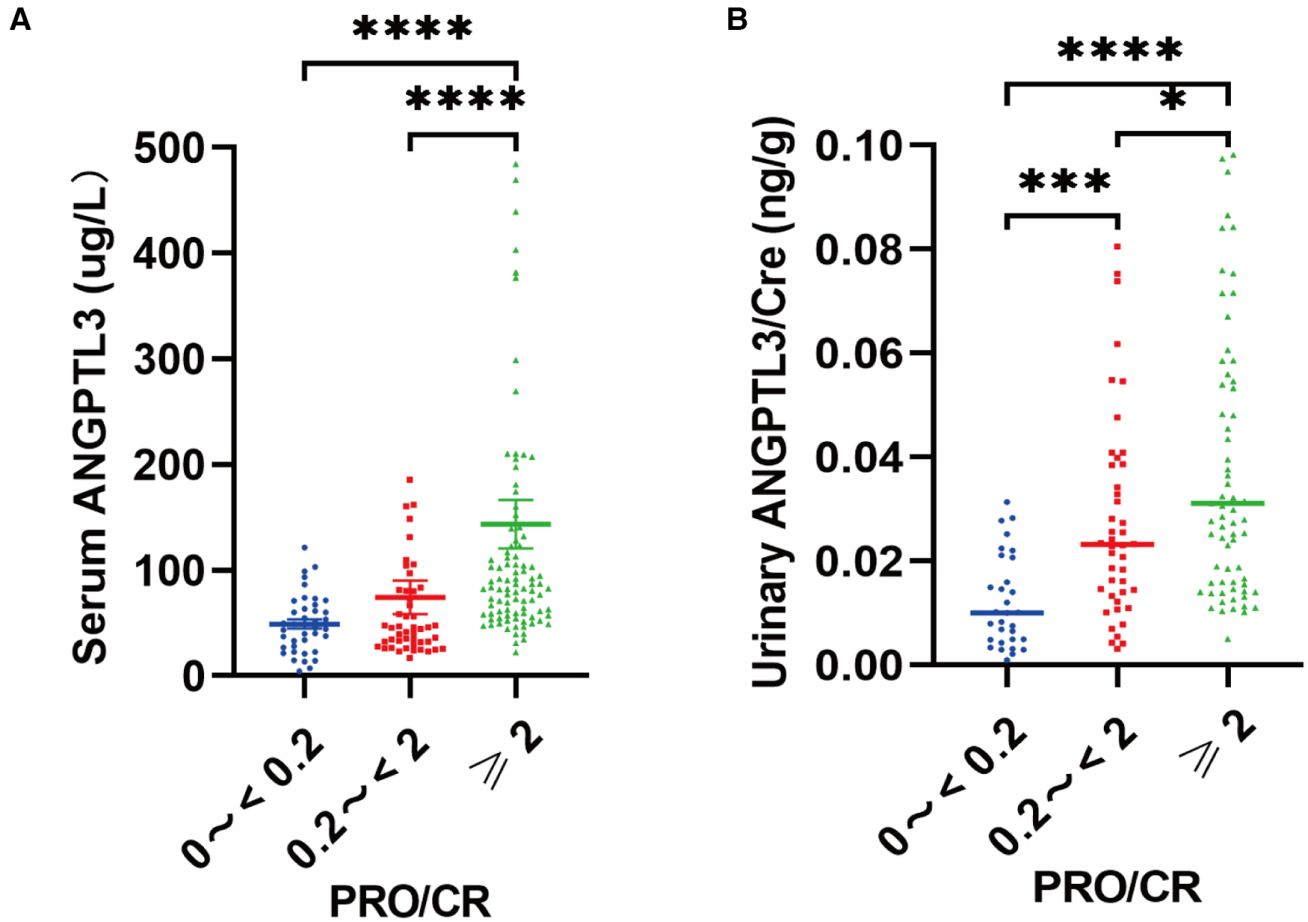
Serum ANGPTL3 and urinary ANGPTL3/Cre were correlated with the degree of proteinuria, and reflected the disease severity of NS. (**A**) The serum samples (*n* = 184) were divided into “0 to <0.2” (*n* = 40), “0.2 to <2” (*n* = 49), “≥2” (*n* = 95) according to PRO/CR. Serum ANGPTL3 was elevated with the degree of proteinuria. (**B**) The urinary samples (*n* = 149) were divided into “0 to <0.2” (*n* = 31), “0.2 to <2” (*n* = 44), “≥2” (*n* = 74) according to PRO/CR. Using Kruskal–Wallis test, *P < 0.05, ***P < 0.001, ****P < 0.0001.

**Figure 4:**
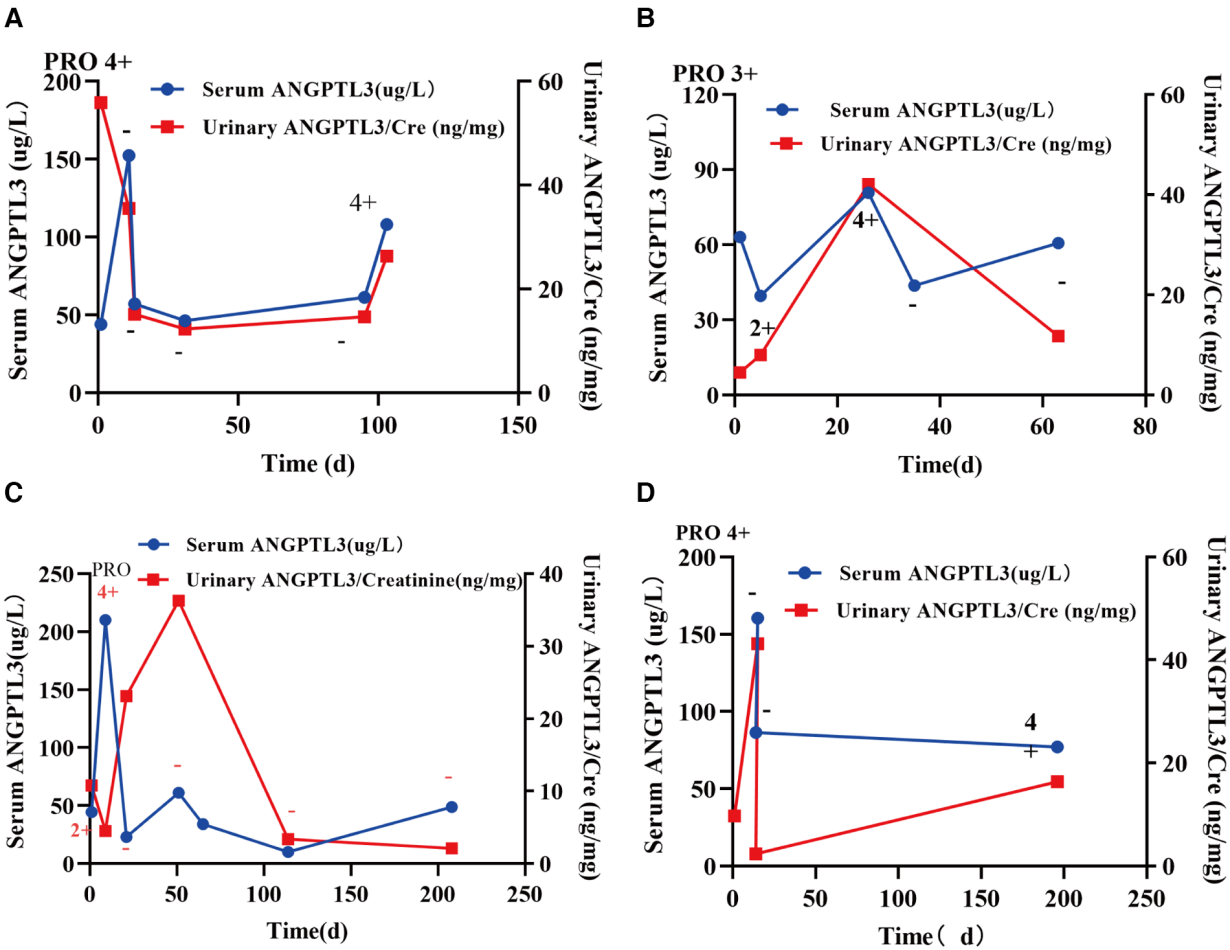
Longitudinal follow-up was conducted in four NS patients at different time points. (**A–D**) The concentrations of serum ANGPTL3 and urinary ANGPTL3/Cre showed almost the same trend as the changes in urinary protein.

We used urine protein level to indicate the severity of nephrotic syndrome disease and grouped NS patients according to urine protein/urine creatinine ratio (PRO/CR) into 3 groups, namely: complete remission: normoproteinuria group: 0 < PRO/CR < 0.2, (0 to <0.2); partial remission: microproteinuric group: 0.2 ≤ PRO/CR < 2, (0.2 to <2); primary/recurrent phase: massive proteinuria group: 2 ≤ PRO/CR, (≥2). Using Kruskal-Wallis test, the results showed that in PRO/CR “0 to <0.2” group vs. “0.2 to <2” group, serum ANGPTL3: 49.165 ± 4.344 µg/L vs. 45.598 (30.756, 80.884) µg/L, *P* > 0.05; urinary ANGPTL3/Cre: 0.0101 (0.0048, 0.0207) ng/g vs. 0.0232 (0.0135, 0.0386) ng/g, *P* = 0.0007; “0 to <0.2” group vs. “≥2” group, serum ANGPTL3: 49.165 ± 4.344 µg/L vs. 84.150 (58.850, 128.250) µg/L, *P* < 0.0001; urinary ANGPTL3/Cre: 0.0101 (0.0048, 0.0207) ng/g vs. 0.0311 (0.0160, 0.0670) ng/g, *P* < 0.0001; “0.2 to <2” group vs. “≥ 2” group, serum ANGPTL3: 45.598 (30.756, 80.884) µg/L vs. 84.150 (58.850, 128.250) µg/L, *P* < 0.0001; urinary ANGPTL3/Cre: 0.0232 (0.0135, 0.0386) ng/g vs. 0.0311 (0.0160, 0.0670) ng/g, *P* = 0.0484 (Figures 3A,B). The results showed that serum ANGPTL3 and urinary ANGPTL3/Cre were significantly elevated during the initial or relapsing phase with massive proteinuria, and serum ANGPTL3 and urinary ANGPTL3/Cre expression decreased during the remission phase when urinary protein decreased and returned to normal. It is suggested that serum ANGPTL3 and urinary ANGPTL3/Cre levels can reflect the degree of urinary protein leakage in patients with nephrotic syndrome and can be used as a marker to assess the severity of nephrotic syndrome in the process of treatment. In addition, longitudinal follow-up was conducted on four NS patients at different time points, and the changes in serum ANGPTL3 and urinary ANGPTL3/Cre levels detected showed almost the same trend as the changes in urinary protein, which suggesting that ANGPTL3 may reflect the disease activity of NS, however, further studies are still needed (Figures 4A–D).

### Serum ANGPTL3 and urinary ANGPTL3/Cre were correlated with clinical characteristics of NS patients

To evaluate the relationship among serum ANGPTL3, urinary ANGPTL3/Cre, and clinical characteristics of NS patients, the spearman correlation analysis was conducted, *r* was used to show the correlation results. The results demonstrated a negative relationship between the serum ANGPTL3 level and age (*r* = −0.2893, *P* < 0.0001), BMI (*r *= −0.1453, *P* = 0.0401), and serum ALB (*r *= −0.5290, *P* < 0.0001). However, serum ANGPTL3 level was positively correlated with the indicators of NS, such as 24 h UPRO (*r* = 0.4892, *P* < 0.0001), PRO/CR (*r *= 0.5039, *P* < 0.0001), TC (*r* = 0.5278, *P* < 0.0001, and TG (*r* = 0.3739, *P* < 0.0001). Consistent with serum ANGPTL3, urinary ANGPTL3/Cre also demonstrated a negative correlation with age (*r* = −0.2257, *P* = 0.0037), serum ALB (*r* = −0.4644, *P* < 0.0001), while exhibited positive correlations with indicators of NS, such as 24 h UPRO (*r* = 0.3949, *P* < 0.0001), PRO/CR (*r *= 0.5363, *P* < 0.0001), TC (*r* = 0.4824, *P* < 0.0001), and TG (*r* = 0.3820, *P* < 0.0001) (Table 2). The levels of serum ANGPTL3 and urinary ANGPTL3/Cre had no statistical difference between male and female (Supplementary Figure S1).

**Table 2:** Correlation analysis among serum ANGPTL3, urinary ANGPTL3/Cre, and clinical variables.

Variables	Serum ANGPTL3 (μg/L)		Urinary ANGPTL3/Cre (ng/g)	
	*r*	*P*	*r*	*P*
Age (month)	−0.2893	<0.0001	−0.2257	0.0037
BMI (kg/m²)	−0.1453	0.0401	−0.0052	0.9478
24hUPRO (g/L)	0.4892	<0.0001	0.3949	<0.0001
PRO/CR (mg/mg)	0.5039	<0.0001	0.5363	<0.0001
Serum ALB (g/L)	−0.5290	<0.0001	−0.4644	<0.0001
TC (mmol/L)	0.5278	<0.0001	0.4824	<0.0001
TG (mmol/L)	0.3739	<0.0001	0.3820	<0.0001

*r*, spearman correlation coefficient; *P* < 0.05 was considered statistically significant.

### Multivariate linear regression analysis indicated that serum ALB was independently correlated with serum ANGPTL3 and PRO/Cr was independently correlated with urinary ANGPTL3/Cre in NS patients

To further figure out the clinical characteristics that may affect the levels of serum ANGPTL3 and urinary ANGPTL3/Cre in NS patients, multivariate linear regression analysis was employed. The results demonstrated that serum ALB was a significant predictor for serum ANGPTL3 (*B *= −4.224, *P* = 0.036) and PRO/CR was independently correlated with urinary ANGPTL3/Cre (*B *= 0.003, *P* = 0.000) (Tables 3, 4). The corresponding multivariate linear regression equations were formulated as follows: *Y* serum ANGPTL3 = −4.224*X1, *X1*: serum ALB; *Y* urinary ANGPTL3/Cre = 0.003*X1, *X1*: PRO/CR.

**Table 3:** Multivariate linear regression analysis was utilized to identify variables associated with serum ANGPTL3 in children with nephrotic syndrome.

Variables	*B*	*P*	95% (CI)	*VIF*
Age (month)	−.085	.772	−.666 to 0.495	1.343
BMI (kg/m²)	2.582	.520	−5.325 to 10.490	1.382
PRO/CR (mg/mg)	1.002	.531	−2.188 to 4.231	1.751
Serum ALB (g/L)	−4.224	.036	−8.172 to −.276	2.728
TC (mmol/L)	−.820	.843	−8.977 to 7.337	2.217
TG (mmol/L)	3.630	.684	−13.953 to 21.212	1.662

Dependent variable: serum ANGPTL3 (μg/L), *P* < 0.05 was considered statistically significant.

**Table 4:** Multivariate linear regression analysis was used to identify variables associated with urinary ANGPTL3/Cre in children with nephrotic syndrome.

Variables	*B*	*P*	95% (CI)	VIF
Age (month)	−4.929 × 10^−5^	.698	.000–.000	1.644
BMI (kg/m²)	.000	.840	−.003 to .004	1.540
PRO/CR (mg/mg)	.003	.000	–.001 to .004	1.744
Serum ALB (g/L)	.001	.455	–.001 to .002	2.926
TC (mmol/L)	.003	.183	−.001 to .007	3.043
TG (mmol/L)	.000	.913	−.007 to .008	1.876

Dependent variable: urinary ANGPTL3/Cre (ng/g), *P* < 0.05 was considered statistically significant.

## Discussion

Angiopoietin-like protein 3 (ANGPTL3) belongs to the angiopoietin family and is a secreted glycoprotein, which was first identified in 1999 and is mainly expressed in the liver and weakly expressed in the kidney (4). In recent years, most studies have focused on ANGPTL3 in lipid metabolism, coronary artery disease atherosclerosis (22–26). However, ANGPTL3 also has made a great progress in the field of kidney disease. In 2001, ANGPTL3 level was firstly found to be upregulated in MCNS patients (15). In the following 20 years, our research mainly focused on ANGPTL3 in aspect to glomerular podocyte injury and proteinuria. Our group discovered that ANGPTL3 was presented in cytoplasm of podocytes of glomeruli and tubular epithelial cells of rat kidney, ANGPTL3 level was found to be upregulated, and it was correlated with proteinuria and hyperlipidemia in ADR-induced nephrotic rats (27). Furthermore, its protective effects on rats with ADR-induced nephropathy were validated by knockdown of ANGPTL3 or anti-ANGPTL3 monoclonal antibodies. In the present study, ANGPTL3 was identified as a diagnostic marker and an indicator for the disease severity of NS. To our knowledge, this is the first study to evaluate the role of ANGPTL3 level in pediatric patients with different kinds of NS children. We all know in clinical practice, the criteria for the diagnosis of nephrotic syndrome are edema, massive proteinuria, hypoproteinemia, and hyperlipidemia (28). Proteinuria is the main clinical manifestation of nephrotic syndrome and is closely related to the progressive deterioration of renal function (29, 30) and previous findings showed that ANGPTL3 was correlated with both hyperlipidemia and proteinuria (31). Also, we analyzed clinical indicators associated with nephrotic syndrome separately and we found that ANGPTL3 level in serum and urine was positively correlated with TC level, TG level, PRO/Cre, 24 h UPRO level and negatively correlated with serum albumin in NS patients, as expected from our results. Multivariate linear regression analysis further demonstrated that serum ALB was independently correlated with serum ANGPTL3 and PRO/CR was independently correlated with urinary ANGPTL3/Cre in NS patients, and indicated that ANGPTL3 could be an alternative indicator for the progression of NS except for proteinuria, which has the advantages of being simpler, faster, and less subject to external factors than 24 h UPRO, and is more suitable for children. Collectively, ANGPTL3 is a key and novel target in NS children. However, this study only confirmed that ANGPTL3 was correlated with proteinuria, whether ANGPTL3 would be the cause or the consequence of proteinuria remained elusive. It has been demonstrated that the integrity of podocytes plays a core role in the proteinuria (32). In recent years, our group concentrated on the role of ANGPTL3 in podocyte injury. ANGPTL3 promoted the motility and permeability of podocytes, and exhibited to play a crucial role in ADR-treated podocyte injury (21). ANGPTL3 was reported as a critical molecular target for cytoskeletal rearrangement of the podocyte by the ADR-induced nephropathy (33). Deletion of ANGPTL3 attenuated proteinuria, and protected podocytes from injury in a mouse model of ADR-induced nephropathy (20). ANGPTL3 knockout not only ameliorated ADR-induced nephropathy in the early stage, but also protected it from progression (19). Furthermore, another study demonstrated that ANGPTL3 activated podocyte-expressed integrin β3 and α-actinin−4, which may result in the cytoskeletal rearrangement of podocytes, depressing ANGPTL3 or interfering with the ANGPTL3-integrin β3 interaction might be benefit for podocyte protection (34). Additionally, Angptl3 knockout alleviated the apoptosis of podocytes by regulating the ROS/GRP78 signaling pathway in lipopolysaccharide (LPS)-induced acute kidney injury (35). In addition to the role of ANGPTL3 in podocyte injury, Angptl3 can also increase the permeability of glomerular endothelial cells and participate in the development of proteinuria *in vitro* (34).

We found that serum and urinary ANGPTL3 combined have a good clinical diagnostic ability for diagnosing the occurrence of NS when their concentrations are higher than 78.940 µg/L and urinary ANGPTL3/Cre concentrations are higher than 0.0101 ng/g. However, when serum and urine ANGPTL3 concentrations are below these thresholds, there are some limitations for differentiating NS. The results of this study are still important for clinical reference. Another, we strictly included the study subjects according to the inclusion and exclusion criteria, collected serum and urine specimens, and measured both serum and urine expression levels of ANGPTL3 according to the strict assay method, and the data were reliable and valid, and the findings provided important and novel information, but due to the study was a single-center, small sample size study, as well as the fact that we did not measure the concentrations of the N and C-terminal fragments of ANGPTL3, hence also subject to limitations. Therefore, whether ANGPTL3 is an useful biomarker in a real clinical setting remains to be further analyzed. At present, we only have demonstrated that the concentrations of ANGPTL3 in serum and urine are significantly higher than those in healthy children. And in patients with nephrotic syndrome, urinary ANGPTL3 showed better sensitivity than serum ANGPTL3, consider that serum ANGPTL3 is not only related to renal podocyte injury but is also influenced by the liver, urinary ANGPTL3 is mainly directly related to renal podocyte injury. However, the combination of the serum and urinary ANGPTL3 has a good clinical adjunctive diagnostic value for the diagnosis of NS, and can assess the severity of nephrotic syndrome disease.

### Strengths and limitations

As far as I know, the greatest strength of our article is that for the first time, blood and urine specimens from nephrotic syndrome due to different glomerular diseases were included to detect the expression level of ANGPTL3. The use of ANGPTL3 as a marker to assess the severity of nephrotic syndrome, based on previous basic experiments, back to clinical patients, provides some theoretical basis for future targeted therapy in patients with proteinuria in nephrotic syndrome. However, our study also has some limitations. First, due to objective factors such as COVID-19 and time, there were some difficulties in collecting specimens from patients and controls, we collected only single-center specimens. Second, the correlation between the levels of ANGPTL3 in serum and urine and the levels of ANGPTL3 in renal tissues of patients with nephrotic syndrome was not tested, and the relationship between ANGPTL3 in serum and urine with renal podocyte injury in patients having nephrotic syndrome was more directly analyzed. Third, no more prospective studies have been performed to analyze the sequence of ANGPTL3 in serum and urine with the activity of renal disease and with the appearance of proteinuria, and whether ANGPTL3 can be used as a marker to predict disease incidence and renal podocyte injury in advance remains to be further explored. In future studies, will need to include more participants and follow-up cohorts through a multi-center, large sample size study to validate the clinical value of ANGPTL3 in clinical NS patients. The future goal is to use podocytes as target cells and ANGPTL3 as a target molecule, to apply anti-ANGPTL3 antibodies or drugs in the clinic to become a new effective target molecule for the treatment of patients with renal podocyte-injured proteinuria.

In conclusion, we provide the first evidence that serum and urine ANGPTL3 level was upregulated in NS patients. Also, ANGPTL3 level in serum and urine was correlated with proteinuria. And revealed that ANGPTL3 level in serum and urine was correlated with the degree of proteinuria and was a valuable marker to assess the disease severity of NS, which showed a satisfactory performance for NS and provided some theoretical basis for the use of anti-ANGPTL3 drugs or antibodies for NS patients in the future clinical translation stage and further exploration of the role of ANGPTL3 may shed light on the treatment of NS.

## Data Availability

The original contributions presented in the study are included in the article/**Supplementary Material**, further inquiries can be directed to the corresponding author.
