# Supplementary material for: Angiopoietin-like protein 3: a novel potential biomarker for nephrotic syndrome in children
Source: Front Pediatr. 2023 May 17;11:1113484. doi: 10.3389/fped.2023.1113484 (PMC10229790; doi:10.3389/fped.2023.1113484)
Supplement: Supplementary file 1 [file Image1.pdf]

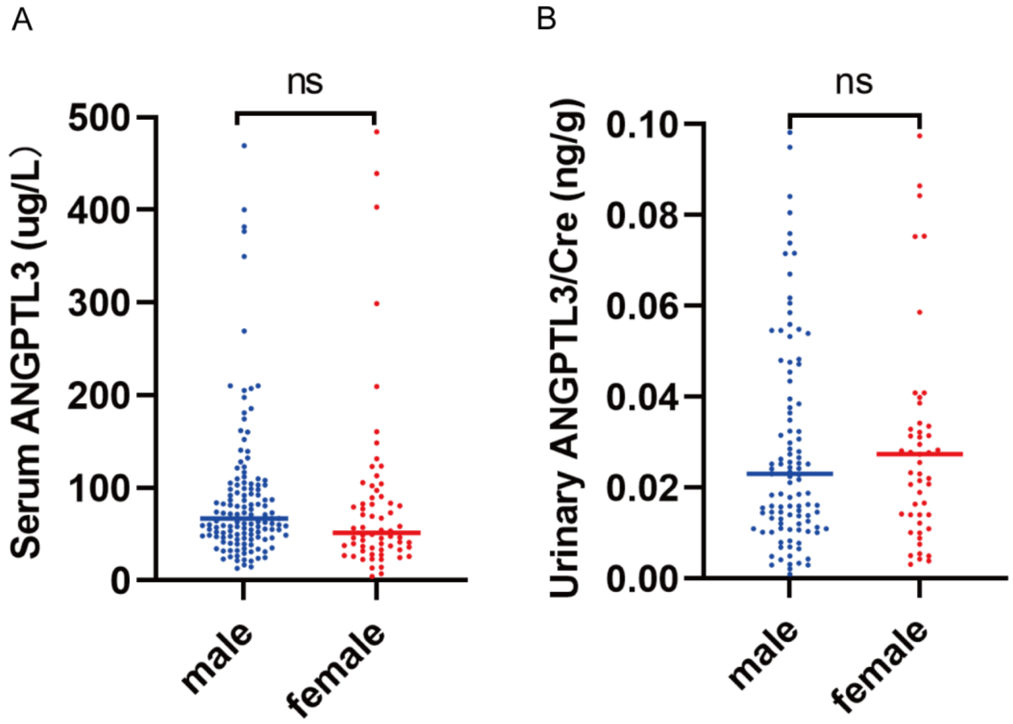

Figure S1 The expression levels of serum ANGPTL3 and urinary ANGPTL3/Cre in male and female NS patients. Using Mann-Whitney U test. ns: no statistically significant difference.
